# Decreased Oxygen Transfer Capacity of Erythrocytes as a Cause of 5-Fluorouracil Related Ischemia

**DOI:** 10.3390/molecules14010053

**Published:** 2008-12-28

**Authors:** Ivan Spasojević, Svetislav Jelić, Joanna Zakrzewska, Goran Bačić

**Affiliations:** 1 Institute for Multidisciplinary Research, University of Belgrade, Kneza Višeslava 1, Belgrade, 11000, Serbia; E-Mail: ivan@cms.bg.ac.yu; 2 National Cancer Research Institute, Pasterova 14, Belgrade, 11000, Serbia; E-mail: jelics@ncrc.ac.yu; 3 Insitute of General and Physical Chemistry, Studentski trg 12-16, Belgrade, 11000, Serbia; E-mail: jzakrzewska@iofh.bg.ac.yu; 4 Faculty for Physical Chemistry, University of Belgrade, Studentski trg 12-16, Belgrade, 11000, Serbia

**Keywords:** 5-Fluorouracil, Cisplatin, Ischemia, Erythrocytes, ^31^P NMR spectroscopy.

## Abstract

Various mechanisms have been proposed to account for chemotherapy related ischemia, but none of them can explain the available clinical data. In order to explore the possibility that the decreased ability of erythrocytes to deliver oxygen to the heart could be responsible for cardiotoxicity, we have performed an *ex vivo* and *in vivo* study of the effects of cisplatin/5-FU on erythrocytes, using a variety of biophysical techniques. Combining EPR and microscopy it was concluded that both cardiotoxic 5-FU and non-cardiotoxic cisplatin have similar effects on the erythrocyte membrane, thus eliminating those changes as a potential source of cardiotoxicity. On the contrary, ^31^P-NMR and polarography showed that the effects of these cytostatics on the intracellular milieu differ significantly. 5-FU provoked a pronounced decrease of the O_2_ level in blood and affected the metabolism of phosphate compounds, while cisplatin had no such effects. When combined these two drugs showed synergistic effects, which matches the higher frequency of cardiotoxicity of the combination relative to the sole application of 5-FU. Preliminary results acquired on blood of patients receiving cisplatin/5-FU therapy verified observations obtained *ex vivo.* These results open a possibility of applying NMR in preclinical trials of new drugs in order to predict their ischemic potential.

## Introduction

Cardiotoxicity of antineoplastic drugs with a possible fatal outcome is a frequent detrimental consequence of chemotherapy [1,2,3]. Since it places limits on the application of chemotherapy and represents a threat to a patient’s health, the cardiotoxicity of antineoplastic drugs has been the subject of numerous clinical studies dealing with various manifestations, such as ischemia, arrhythmia, tachycardia, myocardial infarction, and others [1,2,3,4,5,6,7]. However, ideal monitoring techniques for cardiotoxicity, upon which efficient prophylaxis depends, require a currently lacking thorough understanding of particular biochemical and biophysical, pathophysiological mechanisms of the effects of anti-cancer drugs provoked on the cellular level, which can only be assessed in appropriate *ex vivo* studies [8]. 

5-Fluorouracil (5-FU), which has been in the focus of our previous studies [9,10,11], represents an antineoplastic drug showing frequent cardiotoxic effects [4,5,6,7, 12, 13]. Reported incidence of 5-FU induced cardiotoxicity varies widely between 1.2 and 18%, depending on the application procedure, doses and other parameters [4, 5, 12, 13]. The main symptom of 5-FU cardiotoxicity is ischemia with a resolution time from 20 minutes (acute ischemia) to 2 days (“late events”) [2,3,4,5], and with a wide spectrum of clinical manifestations ranging from silent ECG deviations to myocardial infarction and sudden death [2,3,4,5]. Potential mechanism(s) of 5-FU related cardiotoxicity have been the subject of a number of studies, usually just increasing the pool of case reports [4, 5], but the pathogenesis remained unsolved over more than 30 years from the first report on this issue [7]. For example normal levels of cardiac enzymes and troponin I during episodes of 5-FU-related cardiotoxicity, showed that direct effects of 5-FU on the heart are not the principal cause of cardiotoxicity [5, 6, 14]. Also, it was demonstrated using angiography, that 5-FU cardiotoxicity could not be explained by increased susceptibility to vasospasm [2, 6]. 5-FU has not induced thrombus formation in experimental animals [15], nor have any significant changes of the level of fibrinopeptides in the blood of the patients treated with 5-FU been determined [16]. However, one study offered an alternative concept, proposing that ischemia provoked by 5-FU originates from changes of rheological properties of erythrocytes and consequent increase of blood viscosity [17]. This hypothesis was questioned by an *in vivo* study showing that 5-FU induces a decrease rather than increase of blood viscosity [18]. To resolve such ambiguity, we have expanded the exploration of the *ex vivo* effects of 5-FU on erythrocytes and determined that: (i) 5-FU provokes partially irreversible echinocytosis [10, 11]; (ii) 5-FU increases fluidity of the erythrocyte membrane [11], which could explain the decrease of blood viscosity during 5-FU infusion observed *in vivo* [18]; (iii) 5-FU provokes an efflux of K^+^ [11], which could explain arrhythmia [19], the second most frequent symptom of 5-FU related cardiotoxicity [5]; (iv) 5-FU induces rapid depletion of O_2_ and an increase of the level of 2,3-biphosphoglycerate (2,3-BPG), which decreases the affinity of hemoglobin (Hb) for O_2_ in erythrocytes [20]; and finally (v) 5-FU provokes a significant decrease of the level of ATP [9], which could represent the cause of changes in morphology and ionic balance on the erythrocyte membrane. All these changes affect the normal functioning of erythrocytes that could lead under *in vivo* conditions to insufficient supply of oxygen and ischemia of the heart.

In the present study, we extended our previously used combined ^31^P-NMR/polarography approach [9] to examine the effects of: i) the non-cardiotoxic antineoplastic drug cisplatin in order to compare them with the effects of 5-FU; ii) the therapeutic combination of cisplatin with 5-FU, which is known to be more cardiotoxic (incidence 15-18% [21, 22]) than 5-FU applied alone (up to 3% for bolus application [5, 23]); and iii) *in vivo* effects of 5-FU/cisplatin combination on the blood of oncological patients. It should be noted here, that although cisplatin itself has been ascribed to provoke some cardiac events, these were not, to the best of our knowledge, directly related to cisplatin, but rather represent a consequence of simultaneous application of some other cardiotoxic drugs in combination with cisplatin, or other treatment-related cardiovascular risks [24]. Effects of 5-FU, cisplatin, and cisplatin/5-FU combination on *pO_2_* and on the level of deoxyHb, 2,3-BPG, and ATP in erythrocytes, were correlated with the previously reported frequencies of cardiotoxic manifestation during chemotherapy. Investigation of blood samples and ECGs obtained from patients treated with cisplatin/5-FU therapy was performed in order to compare *ex vivo* determined changes with *in vivo* manifestations.

## Results

**Figure 1:**
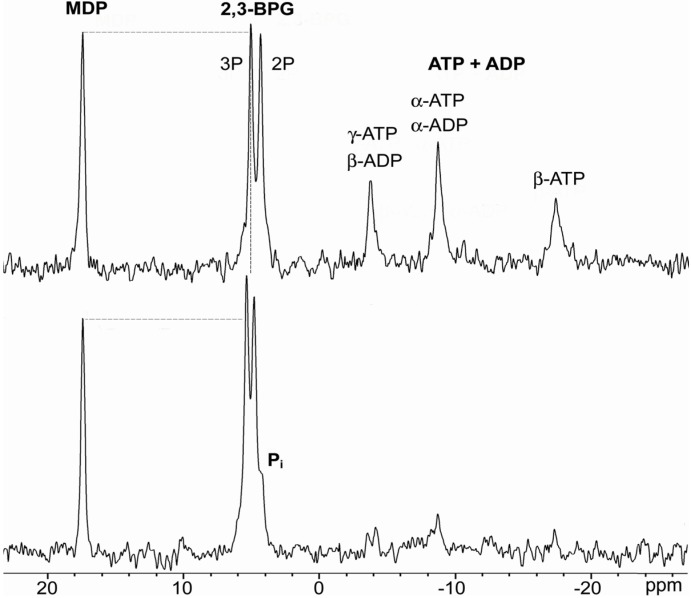
^31^P-NMR spectra of packed erythrocytes after 120 minutes of incubation. Top spectrum: fully oxygenated blood (*pO_2_* = 120 mm Hg); Bottom spectrum: erythrocytes exposed to 10 mg/mL 5-FU. The vertical dashed line marks the change of the chemical shift induced by 5-FU. The horizontal dashed lines demonstrate the increase in the intensity of the 2,3-BPG signal in treated erythrocytes relative to the peak of the MDP standard. The decrease of the ATP level in treated erythrocytes is obvious and pronounced.

Figure 1 contains the essential information on the effect of drugs on erythrocytes but before proceeding to analysis of ^31^P-NMR data it is necessary to analyze other potential causes of cardiotoxicity and to establish the likely sequence of events.

**Table 1:** Effects of cisplatin and 5-FU on the morphology and fluidity of erythrocyte membranes (n=3). Percentage of echinocytes, including all four types, in the total erythrocyte population is presented. In control samples echinocytes type I were observed, while in treated samples type II predominated. Changes of membrane fluidity are described through the EPR order parameter (*S*) which is reciprocally related to the fluidity. *P*<0.05 shows a statistically significant difference between results obtained for treated samples and control samples.

Incubation period (h)		1	2
**Echinocytes (%)** **± S.D.**	Control	5 ± 1	4 ± 3
	Cisplatin	25 ± 7 (*P*=0.005)	36 ± 15 (*P*=0.022)
	5-FU	38 ± 7 (*P*=0.014)	45 ±12 (*P*=0.020)
**Order parameter** ***S* ± S.D.**	Control	0.692 ± 0.007	0.691 ± 0.001
	Cisplatin	0.658 ± 0.016 (*P*=0.026)	0.660 ± 0.005 (*P*<0.001)
	5-FU	0.665 ± 0.012 (*P*=0.032)	0.667 ± 0.010 (*P*=0.014)

Table 1 shows the effect which cisplatin and 5-FU produce on the membrane of erythrocytes in blood exposed to these two drugs. Both provoked the exposure-time dependent transformation of erythrocytes into the echinocytic shape. Cisplatin predominantly provoked formation of echinocytes type I, while 5-FU led to the formation of echinocytes type II. For example after the first 60 minutes of exposure 5-FU led to the formation of types I and II in the 30%:70% ratio, while cisplatin provoked exclusively the formation of echinocytes type I (100%). Nevertheless, differences between the total number of echinocytes were not statistically significant. Consequently, changes in rheological properties of erythrocytes cannot be responsible for the different cardiotoxicity of these drugs.

EPR results (Table 1) further support the above line of reasoning. Both drugs induced significant increase of membrane fluidity. Similar results have been reported previously for 5-FU *in vivo* [18], and explain why provoked echinocytosis could not lead to increased blood viscosity. Although flow of echinocytes through small capillaries of coronary circulation could be hampered due to the altered shape of erythrocytes, increased membrane fluidity points to enhanced flexibility of erythrocytes, which should diminish potentially negative effects of drug induced changes of erythrocyte morphology. Regardless, both drugs (non- and cardiotoxic ones) induce almost the same modification of mechanical properties of erythrocytes, hence showing that changes on the membrane mechanical properties cannot be the principal cause of the chemotherapy related ischemia.

**Figure 2:**
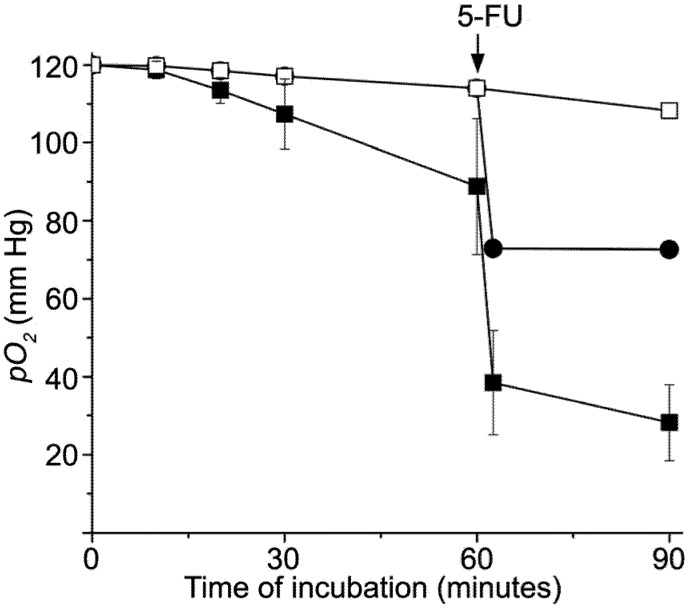
Changes in *pO_2_* induced by *ex vivo* treatment with cisplatin/5-FU. (□) Control samples (n=9); (●) Erythrocytes untreated during the first 60 min of incubation, after which 5-FU (5 mg/mL) was added (n=3); (■) Erythrocytes incubated with cisplatin (0.1 mg/mL) for 60 min, after which 5-FU (5 mg/mL) was added (n=3); Cisplatin provoked statistically significant decrease of *pO_2_* after 60 minutes of incubation (*P*=0.041). After 90 minutes *pO_2_* values in treated samples were significantly different when compared to the control (*P*<0.001). Individual effects of 5-FU were also statistically different when compared to the effects of cisplatin/5-FU treatment (*P*<0.001).

Figure 2 shows only a slight decrease of *pO_2_* in untreated erythrocytes (control) during 90 min of incubation, in contrast to treated samples. Cisplatin induced gradual decrease of oxygen tension so that after 60 minutes of incubation, *pO_2_* was decreased by 30 mm Hg relative to the control. Application of 5-FU led to a more pronounced, and almost immediate decrease of *pO_2_* in untreated erythrocytes (41 mm Hg, also see [9]). 5-FU induced a slightly higher decrease of *pO_2_* in cisplatin pretreated samples (46 mm Hg). The overall effect is serious decrease of *pO_2_* by 76 mm Hg in erythrocytes treated with the cisplatin/5-FU combination. Observed effects are in accordance with higher frequency of cardiotoxic manifestation reported for the cisplatin/5-FU combination [21, 22], than for individual application of 5-FU [5, 23], showing that some synergy could exist. In addition, it is obvious from Figure 2 that biochemical mechanisms of the action of these two drugs on the *pO_2_* in erythrocytes are different.

**Figure 3:**
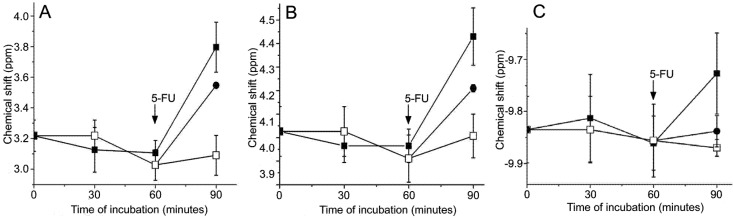
Effects of cisplatin/5-FU on the chemical shift in ^31^P NMR spectra of *ex vivo* treated erythrocytes (n=4). (A) the chemical shift of 2P; (B) and 3P of 2,3-BPG; (C) the chemical shift of α-phosphate in ATP. (□) Control sample; (●) Erythrocytes untreated during the first 60 min of incubation, after which 5-FU (5 mg/mL) was added; (■) Erythrocytes incubated with cisplatin (0.1 mg/mL) for 60 min, after which 5-FU (5 mg/mL) was added. Chemical shift of 2P and 3P signals of treated samples after 90 minutes were significantly different when compared to the control. The effect of 5-FU is also statistically smaller when compared to the effect of cisplatin/5-FU treatment (*P*<0.001). Chemical shift of α-phosphate in ATP obtained from cisplatin/5-FU treated erythrocytes was significantly different from the control (*P*=0.025) and from the shift observed in erythrocytes treated only with 5-FU (*P*=0.031).

^31^P-NMR spectroscopy (Figure 1) is a versatile tool which simultaneously offers information about the level of deoxyHb, 2,3-BPG, and ATP, as well as pH changes, thus providing direct insight into biochemical mechanisms responsible for the altered management of oxygen by erythrocytes under the influence of cytostatics. Figure 3A and Figure 3B show changes in the chemical shift of 2P and 3P signals of 2,3-BPG. Changes in the NMR chemical shift of phosphates in erythrocytes usually result from the changes in magnetic susceptibility due to the appearance of paramagnetic species, such as deoxyHb and/or from pH changes [9, 25]. Deoxygenation and subsequent increase of the amount of deoxyHb is always followed by the increase of the pH value, due to the acid/base properties of deoxyHb [25, 26], hence these two effects are difficult to resolve since both processes produce a downfield shift. However, in this case the observed chemical shift could not originate only from the increased intracellular pH value, since the changes of the shift of 2,3-BPG lines were 2 to 4 times larger than the maximum possible downfield shift induced by the increase of pH related to complete deoxygenation (for a detailed explanation see [9, 25]). Hence, the increase of the concentration of deoxyHb has to be the principal cause for the observed chemical shifts. This is further demonstrated by the significant downfield shift (0.11 ppm) of the α-ATP peak (Figure 3C). Namely, the chemical shift of the α-ATP peak is practically pH independent [25], and can only be produced by changes in the bulk magnetic susceptibility produced by the increased level of deoxyHb.

Data in Figure 3 confirm the effect of two drugs on the oxygen level in erythrocytes but also should allow a quantification of their effect on the oxygenation of erythrocytes. Cisplatin itself did not induce a significant shift in ^31^P-NMR signals of phosphorous compounds within erythrocytes in accordance with Figure 2. However, subsequent application of 5-FU led to a considerable chemical shift of 2P (0.69 ppm), 3P (0.47 ppm), and α-ATP signal (0.11 ppm). Different levels of changes of the chemical shift of these three phosphates in Figure 3 are in accordance with the previously reported relative sensitivity of their chemical shift to deoxygenation of the blood (rank of order: 2P>3P>α-ATP) [25]. Using calibration curves which correlate the shift of 2P and 3P to *pO_2_* given previously by Labotka [25], the decrease of the oxygen tension of blood was calculated to be ~ 90 mm Hg (from 120 mm Hg in fully oxygenated, untreated blood, to 30 mm Hg in treated samples). Changes of the chemical shift provoked by the application of 5-FU to untreated erythrocytes were less pronounced: (0.52 ppm for 2P and 0.3 ppm for 3P peak), which corresponds to the decrease of *pO_2_* in blood of ~ 70 mm Hg. Changes of *pO_2_* evaluated by ^31^P NMR (90 and 70 mm Hg) were higher than in direct polarographic measurements (76 and 47 mm Hg). Such discrepancy can be attributed to a concomitant increase of pH related with acid/base properties of deoxyHb, which only affects evaluation of *pO_2_* by NMR [25]. We used the observed difference, and previously presented calibration curve [25], to evaluate the chemical shift related with changes in pH provoked by 5-FU and cisplatin/5-FU. It has been previously presented that the characteristic shift of ^31^P-NMR signals in erythrocytes due to pH changes is 1.15 and 1 ppm/pH unit for 2P and 3P, respectively [25]. Calculus showed that 5-FU provoked a total increase of pH by 0.09 units, while cisplatin/5-FU *ex vivo* treatment led to an increase of 0.15 pH units.

**Figure 4:**
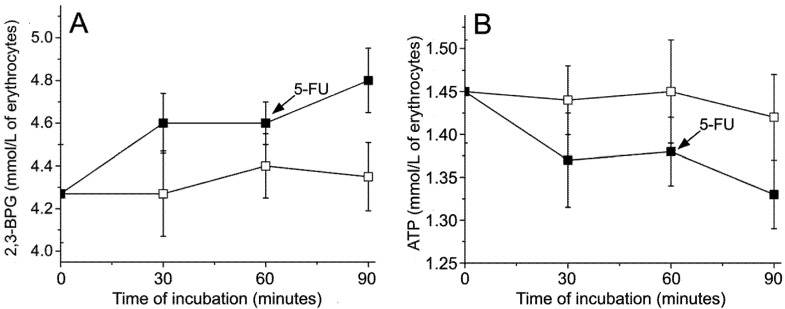
Effects of cisplatin/5-FU on the level of 2,3-BPG and ATP in *ex vivo* treated erythrocytes (n=4). (A), 2,3-BPG; (B) ATP. (□) Control sample; (■) Erythrocytes incubated with cisplatin (0.1 mg/mL) for 60 min, after which 5-FU (5 mg/mL) was added. Increase of the 2,3-BPG level observed after the application of 5-FU was significantly different from control (*P*=0.040), while decrease of the level of ATP was not (*P*=0.061). Cisplatin did not provoke significant changes during first 60 minutes of incubation.

Figure 4A shows that cisplatin provoked only a slight (statistically non-significant) raise of the 2,3-BPG level, which was further increased by the application of 5-FU (statistically significant). Increased level of 2,3-BPG stabilizes deoxyHb [26], and further promotes deoxygenation. The level of ATP in erythrocytes decreased during the application of cytostatics (Figure 4B) indicating the disturbance of the cellular metabolism. Applied concentrations of cytostatics, which correspond to clinical doses, provoked less explicit changes in the ATP level than in our previous study dealing with the effects of high concentrations of 5-FU on erythrocytes [9]. So, although results presented in Figure 4B are not statistically significant, they indicate the presence of a previously established phenomenon that the cardiotoxicity of antineoplastic drugs could be related to their effects on the energetic metabolism of erythrocytes [9]. The observed decrease in the ATP level is likely to be associated with the increased production of 2,3-BPG [20], and could be responsible for the changes of the erythrocyte membrane shape and functionality, since the cytoskeleton consumes energy for its functioning [27].

**Figure 5:**
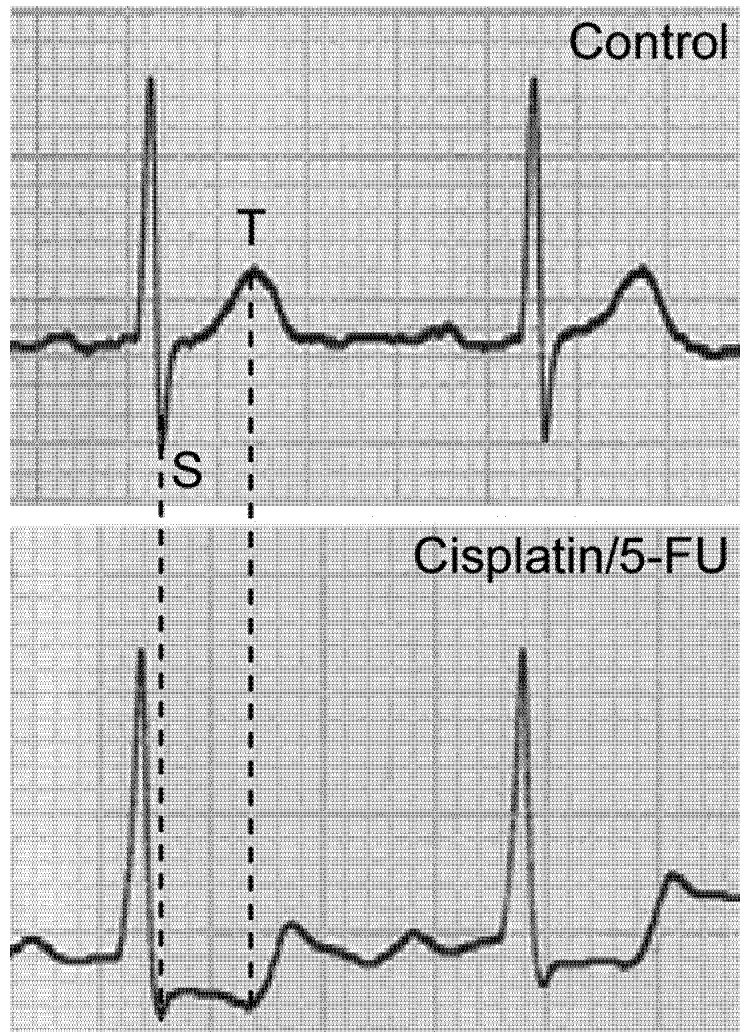
Characteristic electrocardiograms of oncological patients treated with combined cisplatin/5-FU therapy obtained: before the therapeutic cycle (upper trace); and after the bolus application of 5-FU (lower trace).

Patients involved in this study did not have pre-existing heart disease, and their electrocardiographs were normal prior to the therapeutic cycle (Figure 5). ECGs remained normal during cisplatin infusion, but ECGs obtained after the bolus injection of 5-FU showed depression of the ST segment, characteristic for ischemic manifestations. The ECG changes presented in Figure 5 were observed in 3 patients, they were slightly less pronounced in one, while one showed no changes.

**Table 2:** Degree of the shift of signals of phosphates in the blood of patients (n=5) treated with combined cisplatin/5-FU therapy. Results are presented as the difference between mean values of the chemical shift of phosphates signals in the blood samples obtained before the therapy and after the application of 5-FU (± standard deviation). Infusion of cisplatin itself did not provoke significant changes.

**^31^P-NMR signals**	2P (2,3-BPG)	3P (2,3-BPG)	α-ATP
**Shift (ppm)**	0.051 ± 0.037 (*P*=0.981)	0.042 ± 0.037 (*P*=0.976)	0.061 ± 0.014 (*P*=0.618)

Application of 5-FU after cisplatin in all studied cases led to a downfield shift in ^31^P-NMR spectra of blood samples obtained from patients (Table 2). The number of patients in the clinical study was small, so adequate statistical analysis could not be performed. Nevertheless, two important conclusions can be extracted from these preliminary results: first, the presented trend in the results indicates that deoxygenation observed *ex vivo*, also occurs under *in vivo* conditions; and second, ^31^P-NMR can be used to track *in vivo* changes in blood during the course of therapy, providing vital information about the level of O_2_ in blood, which could not be obtained by less sensitive pulsed oxymetry.

## Discussion

Various concepts of pathogenesis of 5-FU related cardiotoxicity have been proposed [4, 5], but without sufficient support from biochemical studies. Levels of cardiac enzymes and troponin I, that have been reported to remain normal during episodes of 5-FU-related cardiotoxicity, show that direct effects of 5-FU on the heart are not the principal cause of cardiotoxicity [5, 6, 14]. It has also been demonstrated using angiography that 5-FU cardiotoxicity could not be explained by increased susceptibility to vasospasm [2, 6]. 5-FU has not induced thrombus formation in experimental animals [15] nor have any significant changes of the level of fibrinopeptides in the blood of the patients treated with 5-FU been determined [16]. Hence it is not surprising that changes on erythrocytes such as modifications of rheological properties of erythrocytes and consequent increase of blood viscosity have been proposed as a cause of 5-FU provoked ischemia [17]. We show here that cardiotoxic 5-FU and non-cardiotoxic cisplatin induce comparable effects on rheological properties of erythrocytes, such as morphology and membrane fluidity, demonstrating that the principal cause of cardiotoxic effects of these drugs are not the changes of the membrane, but rather those at the intracellular level. 

The polarographic study showed that 5-FU induces rapid depletion of *pO_2_* and that the effect is more pronounced in erythrocytes pretreated with cisplatin. This can trigger the following chain of events. Immediate decrease of *pO_2_* provokes increased production of 2,3-BPG, which leads to further deoxygenation and to the increase of the level of deoxyHb. Increased production of 2,3-BPG most likely represents the cause for the observed decrease in the ATP level. The decreased ATP level should represent the principal cause for the observed changes of membrane rheological characteristics and ionic misbalance. All these changes diminish the ability of erythrocytes to deliver oxygen to the heart.

Measurements of the chemical shift of ^31^P-NMR signals of erythrocytes provided information about the relative level of deoxyHb, which can be directly correlated to cardiotoxic effects during chemotherapy. Non-cardiotoxic cisplatin did not provoke any changes in the level of deoxyHb, while cardiotoxic 5-FU induced a significant increase. When erythrocytes were treated with a cisplatin/5-FU combination, which is known to exhibit higher occurrence of cardiotoxic manifestations than 5-FU alone [21,22,23], even more pronounced deoxygenation was observed. These results, verified by the polarographic study, indicate the synergistic negative effects of 5-FU and cisplatin, as proposed earlier [2]. Non-cardiotoxic cisplatin could amplify the effects of 5-FU on erythrocytes via the previously described interaction of cisplatin with Hb [28], or some of the components of the antioxidative system [29]. In addition, cisplatin itself showed the ability to provoke a slight decrease of *pO_2_*, which under *in vivo* conditions might not lead to detectable ischemia, but could amplify the ischemic effects of 5-FU. 

Preliminary results obtained in the ^31^P-NMR study on the blood of patients receiving combined cisplatin/5-FU therapy, indicate that the combination provokes deoxygenation *in vivo*. Usual total doses of 5-FU for bolus application are 600-1,500 mg [6], so up to 1.5 g of 5-FU is injected at once into the blood stream of the patient. This means that the bolus application leads to temporary exposure of approximately 300 mL of blood to the concentration of 5-FU used in this study. When the significant volume of blood, which has undergone rapid deoxygenation amplified by the prior infusion of cisplatin, enters the coronary circulation, acute ischemia occurs (see Figure 5). 

However, in some cases, especially with 5-FU applied via long term infusion, ischemia has been observed some time after the beginning of chemotherapy [5]. In the light of presented results, this could be explained by less prominent, but prolonged and extensive blood deoxygenation. However, it could be conceived that the bolus application of 5-FU, followed by continuous infusional application of the drug, could prove more deleterious in regard to the drug oxygen-depleting activity than either of the two application modalities administered alone. The reduction of functionality could be induced at a broader population of erythrocytes. It is based on the irreversible decrease of the ATP level and irreversible changes of membrane properties and ionic balance. Presence of non-functional erythrocytes in the blood could contribute to the development of “silent” anemia and chronic ischemic heart conditions. No “true” anemia occurs during 5-FU administration since the drug does not cause hemolysis. Similar mechanisms could potentially explain the frequent neurotoxic effects of anti-neoplastic drugs [30].

Our study was aimed at showing that ^31^P-NMR parameters of erythrocytes correspond to the occurrence of ischemic manifestations. However, further research of the effects of cisplatin, 5-FU and their combination on work capacity and blood flow of the perfused heart model and the study of effects provoked on the wider population of oncological patients are needed in order to more explicitly establish such a correlation. 

^31^P-NMR proved to be a very valuable tool for investigation of mechanisms of cardiotoxicity of antineoplastic drugs, demonstrating excellent correlation with cardiotoxic manifestation observed in clinical practice. However, we do not claim that this is a universal method since changes in O_2_ metabolism do not represent the only known mechanism of chemotherapy related cardiotoxicity. Anthracyclines were reported to provoke such effects through oxidative stress; however we showed that this is not the case with cisplatin/5-FU. Therefore ^31^P-NMR of erythrocytes represents one of the standard methods that should be applied in the investigation of mechanisms of ischemia of a certain class of antineoplastic drugs. Also, it could be applied in preclinical trials of newly developed drugs to determine and anticipate their ischemic potential, thus reducing the usage of experimental animals. Finally, since the reaction of patients to chemotherapy varies considerably and was shown not to be related to a preexisting heart condition, potential strategies of prophylaxis for individual patients could be based on the application of ^31^P-NMR of erythrocytes prior to the treatment with cytostatics that are known to cause cardiotoxicity through the mechanisms outlined in this paper.

## Experimental

### Blood samples

Fresh blood was obtained from five healthy, young volunteers between the ages of 20 and 30, using tubes containing 0.072 mL of 7.5% K_3_EDTA as the anticoagulant per 3 mL of blood (Vacuette EDTA, Greiner Bio-One GmbH, Kremsmünster, Austria). Blood was collected several times from the same participant, according to the needs of a particular experiment. Hematocrit was determined to be ~40% in all samples. Blood was kept at 37°C, immediately after collection or after the washing procedure, depending on the demands of the employed analytical procedure. To establish the relative degree of hemolysis in both controls and treated blood samples, plasma or supernatant (depending on the used procedure) was diluted 10 times and absorbencies at 540 nm were measured to determine the potential release of hemoglobin from erythrocytes [11]. No significant level of hemolysis was observed in any of the treated samples (data not shown).

### Erythrocyte Morphology

Following incubation with cisplatin (final concentration - 0.1 mg/mL) or 5-FU (5 mg/mL), as well as untreated erythrocytes were immediately fixed according to the May-Grünwald Giemsa procedure (dyes were purchased from Sigma-Aldrich, St. Louis, MO, USA). Light microscopy was applied to determine the percentage of echinocytes in the total erythrocyte population. Echinocytes were divided into four types: I – IV, according to the criteria described earlier [31].

### EPR Spectroscopy

Immediately after collection, blood was incubated for 1 or 2 h with cisplatin (0.1 mg/mL) or 5-FU (5 mg/mL). Erythrocytes were washed twice with a NaCl-Tris isotonic buffer (140 mM NaCl, 20 mM Tris, pH adjusted to 7.4 with 1 M HCl) by centrifugation at 3500 x g/10 min/4°C, and re-suspended in the buffer to obtain hematocrit of 40%. Erythrocyte membranes were labeled using 7-DS (2-(5-carboxypentyl)-2-undecyl-4,4-dimethyloxazolidine-3-oxyl; Molecular Probes, Junction City, OR, USA), a nitroxide spin-probe, as described previously [11]. EPR spectra were recorded using a Varian E104-A EPR spectrometer operating at X-band (~9.1 GHz) with acquisition parameters: microwave power, 5 mW and modulation amplitude, 0.2 mT. The order parameter (*S*), which is reciprocally proportional to the membrane fluidity, was calculated as previously described [11]. The EPR spin-trapping technique using DEPMPO (5-diethoxyphosphoryl-5-methyl-1-pyrroline-N-oxide; Alexis Biochemical, Lausen, Switzerland) was applied to investigate whether 5-FU and/or cisplatin provokes generation of free radicals in erythrocytes. It was observed that these drugs do not induce a detectable level of any reactive species (data not shown).

### Oxygen Tension Measurements

A polarographic (Clark-type) electrode (Hansatech Instruments Ltd, King’s Lynn, England) was used for measurements of oxygen tension (*pO_2_*) in blood samples. Collected blood was centrifuged at 3,500 x g/10 min/4^o^C and the supernatant was carefully removed. Erythrocytes were washed twice, re-suspended in a phosphate buffer solution (NaCl 8.8 g/L, Na_2_HPO_4_ 1.2 g/L, NaH_2_PO_4_ 0.43 g/L, pH 7.4) to obtain hematocrit of 40% and incubated for 40 min in humidified air to achieve full oxygenation of 120 mm Hg [25]. After that, the samples were treated as in NMR experiments (see below). Oxygen tension was measured at regular intervals during the 90 min incubation period (see Figure 2). Special attention was paid to the point where 5-FU was added since it has been determined that the application of 5-FU led to rapid decrease of *pO_2_* within the first 2 minutes after application [9]. Before each experiment the electrode was calibrated by exposing fully oxygenated blood to the N_2_ atmosphere to achieve full deoxygenation (from *pO_2_*=120 to 0 mm Hg). 

### ^31^P-NMR Spectroscopy

NMR measurements were performed using a Bruker MSL 400 NMR spectrometer (magnetic field strength 9.41 T). Fresh blood obtained from healthy volunteers was incubated for 40 min in humidified air to achieve full oxygenation (*pO_2_*=120 mm Hg) [25]. Each sample was divided into 3 parts. One was incubated for 90 min and served as a control. The second was incubated without cisplatin for 60 min, after which 5-FU was added (5 mg/mL) and the incubation was 90 min in total. The third was incubated for 60 min with cisplatin (0.1 mg/mL), after which 5-FU was added (final concentration - 5 mg/mL) and incubation was continued for an additional 30 min. *Ex vivo* experiments with such a time course were carried out in order to simulate *in vivo* conditions during standard cisplatin/5-FU chemotherapy, which starts with the infusion of cisplatin, followed by the bolus application of 5-FU [21, 22]. Applied concentrations of 5-FU and cisplatin closely mimicked therapeutic concentrations in blood. Aliquots were taken at every 30 min of incubation. Erythrocytes were packed by centrifugation at 3,500 x g/10 min (final hematocrit value was 70%), and placed in a 10-mm quartz tube capped with a plastic cap to control gas exchange. A capillary containing 25 mM MDP (methylene diphosphonate), was placed in the center of the NMR tube as an external chemical shift (17.05 ppm relatively to 85% H_3_PO_4_) and peak intensity standard [25]. Chemical shift and line width of the reference signal of MDP within the inner tube were not changed during our experiments, which is in agreement with reported lack of dependence of these parameters on the magnetic susceptibility of the solution in the outer tube under similar experimental conditions [32]. Absolute concentration of ATP was determined by measuring the β-ATP peak area, since the other two peaks overlap with signals of ADP (Figure 1). Absolute concentration of 2,3-BPG was determined by measuring the area of the 3P peak since the 2P peak overlaps with the signal of inorganic phosphorus - P_i_ (Figure 1). In order to determine absolute concentrations peak areas were compared to the peak area of MDP, which concentration was known, taking into account individual saturation factors due to different *T_1_* values (for details see [9]). Other experimental conditions were: repetition time 250 ms, pulse angle 40^o^, the signal was accumulated for approximately 16 min (4000 accumulations). Figure 1 illustrates parameters obtainable by ^31^P-NMR of erythrocytes, comparing a characteristic NMR spectrum of packed erythrocytes to the spectrum of erythrocytes treated with 10 mg/mL of 5-FU for 2 hours.

### Patients and Treatments

Blood was obtained from five oncological patients with similar cisplatin/5-FU treatment regimens: cisplatin (80-100 mg/m^2^) was administered in a 2 h infusion, followed by the bolus application of 5-FU (400-750 mg/m^2^). The bolus application was then followed by continuous infusional application as described in the deGramont regimen. Two patients have been diagnosed with gastric cancer, two with cancer of the esophagus and one with head and neck cancer. Blood samples of 5 mL each, were taken before therapy, at the end of cisplatin infusion and following the bolus application of 5-FU. Erythrocytes were packed (hematocrit 70%) and NMR measurements were conducted as described. Electrocardiograms were obtained for all patients before the therapeutic cycle and during the application of 5-FU. Pulse oxymetry of oxygen saturation of arterial blood was performed during the entire course of therapy. All patients were treated at the National Cancer Research Centre, Belgrade, Serbia. Institutional approval for the study was granted by the Ethics Committee in accordance with internationally accepted ethical standards (The Helsinki Declaration of 1964, as revised in 1975, 1983 and 1989). Each patient received adequate explanation and signed the informed consent form.

### Statistical Analysis

The data are presented as the means ± the standard deviation of experiments preformed on 3 or more separate blood samples obtained from different volunteers or patients. Statistical differences between the values obtained on controls and treated samples were evaluated by means of the t-test for independent samples, using Statistica 6.0 (StatSoft Inc, Tulsa, OK, USA). Results were taken to be statistically different if *P*<0.05. 
